# Editorial: Online Mindfulness Intervention Delivery: Efficacy and Adherence

**DOI:** 10.3389/fpsyg.2022.856135

**Published:** 2022-03-03

**Authors:** Susan K. Johnson, Paula Goolkasian

**Affiliations:** Department Psychological Science, University of North Carolina at Charlotte, Charlotte, NC, United States

**Keywords:** online, mindfulness, intervention delivery, efficacy, adherence

## Introduction

Research examining online delivery of mindfulness-based interventions (MBIs) has accelerated during the current COVID-19. Recent studies (Johnson et al., 2014; Forbes et al., 2020) have demonstrated the feasibility of delivering online MBIs aimed at the treatment of chronic illnesses, cancer, psychological disorders, and stress. However, there is a crucial need for a greater understanding of the most efficacious formats, likely benefits, and obstacles to adherence for online MBIs (Carmody and Baer, 2009; Parra et al., 2019). Rates and predictors of adherence have not been systematically investigated in MBIs in general, nor in online delivery in particular. A full understanding of who benefits from MBIs is undermined without understanding their reach. This Research Topic aims to address the efficacy of online delivery of MBIs, in terms of benefits and integrity, as well as revealing obstacles and predictors that affect rates of adherence.

## MBIs

The empirical studies presented in this Research Topic highlight the efficacy of online delivery with a variety of MBIs geared to reduce stress and improve positive health-related behaviors. The interventions ranged from those that are popular and commercially available to those tailored to address specific health-related conditions. For example, Ball and Rivas in an opinion article report on some findings with Headspace, a commercial app. Although easy to use and recommended by health care staff, they find the app underutilized because of technical issues or time constraints due to busy lifestyles. The effectiveness of Headspace is compared with Peak, a cognitive training app, by Haliwa et al. in a 10-day intervention with a college sample. Their results show that 10-min a day mindfulness training was effective at increasing state mindfulness; but cognitive training with 4 daily games and puzzles were equally effective at changing trait mindfulness and ratings of positive and negative mood. Interestingly, their rates of adherence were 90%, much higher than found in other studies.

Some of the online interventions were adapted from in-person MBIs. The standardized 8-week Mindfulness-Based Stress Reduction program (MBSR, Kabat-Zinn, 2013) was adapted into a synchronous online program *via* Zoom by Sanilevici et al. during the first wave of the COVID-19 Pandemic. Findings showed that the invention group showed a decrease in anxiety and stress, and an increase in emotion regulation relative to the control group. Moreover, the effects were consistent in a 1-month follow-up. Klatt et al. compared an 8-week online program during COVID-19 to an in-person pre COVID-19 delivery of a Mindfulness in Motion program. Both groups showed similar pre/post changes in workgroup engagement/resiliency and adherence was 80% or above in both groups.

By contrast, the MBIs used in the other empirical studies in this Research Topic are customized to address specific health or work-related concerns. Mitchell et al. used guided imagery to create 4-min audio files promoting physical activity in a pilot study with underactive adults. Pre/post testing associated with a 2-week intervention showed that the increase in mindfulness was accompanied by an increase in physical exercise and exercise satisfaction. Forbes and Johnson compared adherence rates and effectiveness of an intervention with 4-online self-paced modules directed at patients with Irritable Bowl Syndrome. Adherence rates were higher (44%) for those who received support each week in the form of email reminders when compared to those who did not (11%). Positive outcomes were found, however, with a lower incidence of stress and depression, and an increase in mindfulness and mindful eating. Similar increases in mental health were found by Dorais and Gutierrez when they conducted a 4-week intervention with college students. Each participant used a unique spiritual meditation with a centering prayer for 10 min twice a day. When compared to a wait list control, mindfulness increased and perceived stress and anxiety decreased. Lastly, in a massively open online course directed at a global group of English-speaking participants, a 6-week mindfulness intervention conducted by Bartlett et al. found that mindfulness was associated lower perceived stress and higher work engagement. Both cross sectional and longitudinal data from the pre/post surveys showed strong relationships with mindfulness.

## Conclusion

Taken together, these studies show that delivering interventions online are a way to allow more people to benefit from MBIs; and that for all of the studies, irrespective of type and format of MBIs, the increase in mindfulness due to the intervention is associated with a decrease in perceived stress as well as a number of other health-related behaviors such as anxiety and depression. In addition, Sanilevici et al.'s finding of a mediating effect of MBSR on mental health through emotional regulation suggests that mindfulness-based interventions may be particularly well-suited to treat the physical and psychological manifestations of chronic stress and related illnesses. An additional take away from these studies is the fact that adherence with the online intervention protocol was higher when participation was supported either by group interactions or feedback/incentives from daily/weekly recordings than when it was individual and self-determined.

Since the studies, however, sampled from those who volunteered because of their interest in mindfulness training and were predominately women, the findings cannot at this point be generalized to anyone outside of those groups. Also, pre/post designs were used and although some included a comparison group composed of waiting list controls, only one study compared the results to an active control group. So, although the findings show that online MBIs are promising, there is a need for additional research with representative samples and research designs that allow either in-person comparisons or active controls using random assignment to groups.

## Author Contributions

SJ and PG were the topic editors for the special issue. SJ wrote the aims and scope for the Research Topic. PG wrote the Editorial. All authors contributed to the article and approved the submitted version.

## Conflict of Interest

The authors declare that the research was conducted in the absence of any commercial or financial relationships that could be construed as a potential conflict of interest.

## Publisher's Note

All claims expressed in this article are solely those of the authors and do not necessarily represent those of their affiliated organizations, or those of the publisher, the editors and the reviewers. Any product that may be evaluated in this article, or claim that may be made by its manufacturer, is not guaranteed or endorsed by the publisher.
